# Cuttlefish color change as an emerging proxy for ecotoxicology

**DOI:** 10.3389/fphys.2023.1162709

**Published:** 2023-03-08

**Authors:** Anaïd Gouveneaux, Antoine Minet, Christelle Jozet-Alves, Thomas Knigge, Paco Bustamante, Thomas Lacoue-Labarthe, Cécile Bellanger

**Affiliations:** ^1^ Ethologie Animale et Humaine (EthoS), UMR 6552 CNRS, Université Caen Normandie, Caen, France; ^2^ Stress Environnementaux et Biosurveillance des Milieux Aquatiques (SEBIO), UMR-I 02, Université Le Havre Normandie, Le Havre, France; ^3^ Littoral Environnement et Sociétés (LIENSs), UMR 7266 CNRS-La Rochelle Université, La Rochelle, France

**Keywords:** behavior, body pattern, camouflage, cephalopod, chromatophore, crypsis, mollusk, neurotoxicity

## Abstract

Lately, behavioral ecotoxicology has flourished because of increasing standardization of analyses of endpoints like movement. However, research tends to focus on a few model species, which limits possibilities of extrapolating and predicting toxicological effects and adverse outcomes at the population and ecosystem level. In this regard, it is recommended to assess critical species-specific behavioral responses in taxa playing key roles in trophic food webs, such as cephalopods. These latter, known as masters of camouflage, display rapid physiological color changes to conceal themselves and adapt to their surrounding environments. The efficiency of this process depends on visual abilities and acuity, information processing, and control of chromatophores dynamics through nervous and hormonal regulation with which many contaminants can interfere. Therefore, the quantitative measurement of color change in cephalopod species could be developed as a powerful endpoint for toxicological risk assessment. Based on a wide body of research having assessed the effect of various environmental stressors (pharmaceutical residues, metals, carbon dioxide, anti-fouling agents) on the camouflage abilities of juvenile common cuttlefish, we discuss the relevance of this species as a toxicological model and address the challenge of color change quantification and standardization through a comparative review of the available measurement techniques.

## 1 Introduction

For ethical reasons and given the growing need for risk assessment of environmental pollutants, the field of ecotoxicology is undergoing substantial changes (Campbell et al., 2022). In line with the 3Rs principle (Replace, Reduce, Refine), alternatives are being sought to replace conventional toxicity tests, which frequently measure mortality as an endpoint. Among them, the development of sublethal behavioral endpoints is not new (Dell’Omo, 2002), but attracts increasing attention. Indeed, it has been shown that a variety of behavioral responses to contaminants, both in vertebrates and invertebrates, appear to reflect alterations in sensory, hormonal, neurological, and/or metabolic systems (Saaristo et al., 2018; El-Gendy et al., 2021). Several authors have suggested that behavior could be among the most sensitive, flexible and conspicuous expressions of an animal’s integrated physiological response, making it a suitable endpoint for early toxicity testing (e.g., Clotfelter et al., 2004; Hellou, 2011; Melvin and Wilson, 2013; Peterson et al., 2017).

A large proportion of the research conducted so far in aquatic species has targeted behaviors such as predator avoidance, locomotor activity, exploration and anxiety (Melvin and Wilson, 2013). They are doubtless highly relevant to assess ecological risks as they reflect a wide range of biological functions, including predation, feeding, migration and mating (El-Gendy et al., 2021). Moreover, some behavioral tests using these endpoints allow high-throughput screening assays in laboratories and can be adapted to various species. However, diversifying the approaches, toxicological endpoints and model organisms is necessary (1) to account for inter-specific differences in sensitivity and toxicodynamics and (2) to make extrapolations and predictions of toxicological and ecological effects possible (Segner and Baumann, 2016). In this regard, some critical species-specific behavioral responses must be assessed in species playing key roles in trophic food webs, such as cephalopods. As mollusks—an important phylum among marine invertebrates—they certainly offer a particularly large and rich behavioral repertoire, including remarkable color changes that may serve as a proxy, notably for neurotoxic effects of environmental chemicals (Hanlon and Messenger, 2018).

**FIGURE 1:**
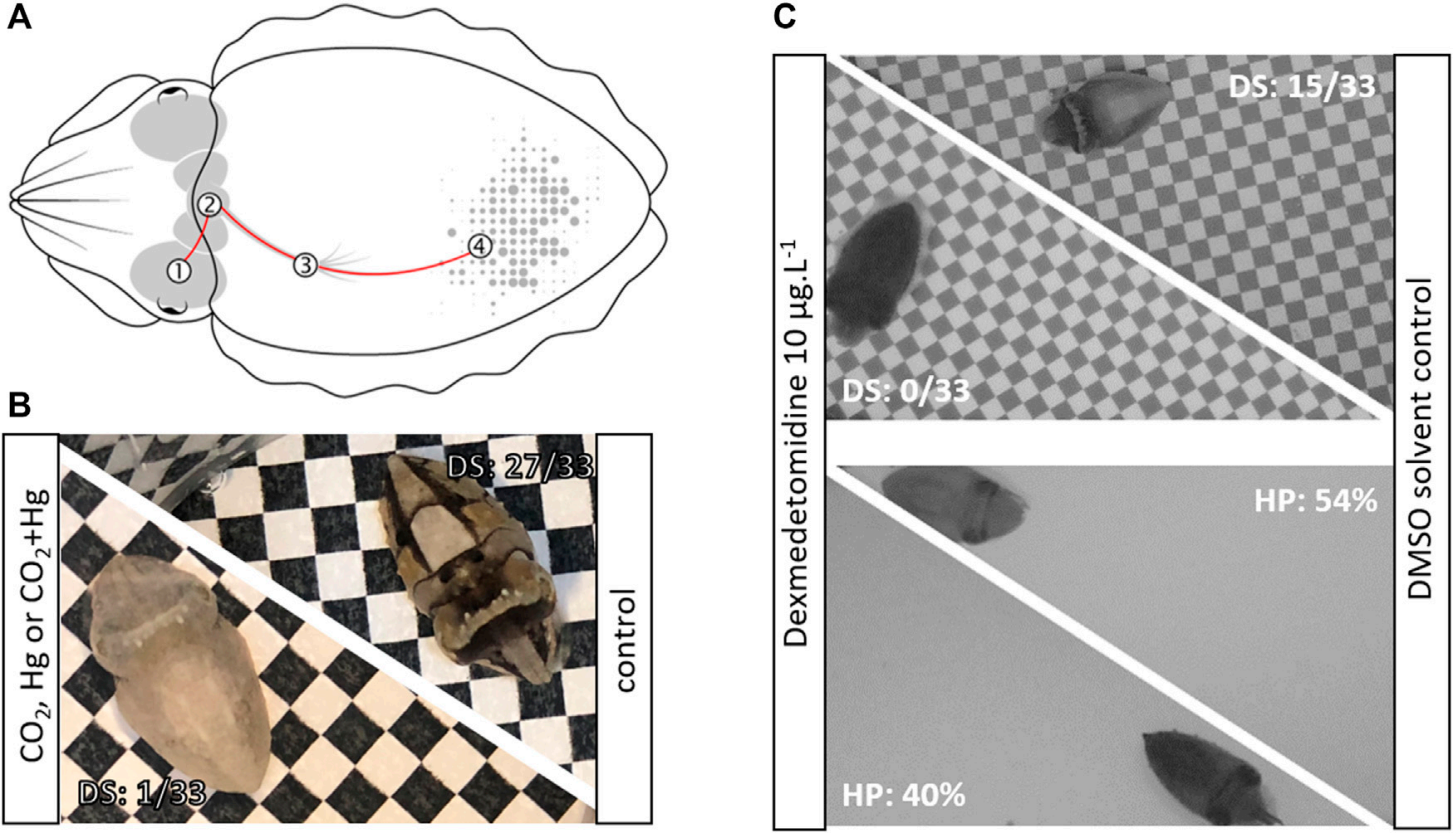
**(A)**. Diagram of the neuronal control of chromatophores in the European cuttlefish: The visual information is transmitted from the eye (1) to the central nervous system (through the optic lobes, the lateral basal lobes, then the chromatophore lobes) that controls the motor activity of chromatophores (4) through the stellate ganglion (3), **(B)**. Example of cuttlefish hatchlings exposed for 1 month to CO_2_ (i.e., pH 7.54), Hg (i.e., 3 μgg^-1^ dry weight in muscle) or CO_2_+Hg, with a low disruptive score (left) compared to a cuttlefish from control conditions (i.e., pH 8.02; right) with a high disruptive score, **(C)**. Example of cuttlefish hatchlings exposed for 3 days to dexmedetomidine during 3 days displaying a dark uniform pattern on both checkerboard and uniform backgrounds compared to cuttlefish from solvent control conditions (DMSO: dimethyl sulfoxide; DS: disruptive score; HP: homochromy percentage).

Color change is a widespread ability among animals, known to fulfill various biological functions such as thermoregulation, UV protection, crypsis or communication (Figon and Casas, 2018). It is generally achieved either by the production, degradation or chemical modification of pigmented structures (morphological color change) or by changes in intracellular pigment distribution within specialized skin organs called chromatophores (physiological color change). In cephalopods, the chromatophores consist of pigment-containing elastic sacculi attached to a set of neuromuscular fibers, whose mechanical action controls the dispersion or the concentration of pigments (Messenger, 2001; Figure 1A). These basic structural *elements* are organized in *units*, themselves organized in chromatic *components*, which, combined with textural (i.e., expression of skin papillae), postural (e.g., arm posture) and locomotor components, form a palette of species-specific *body patterns* (Hanlon and Messenger, 2018).

Multiple experiments have shown that the choice of body pattern relied on a fine visual analysis of the animal’s immediate surroundings, considering, not only the nature of the substrate, but also the presence of objects, conspecifics, prey or predators (Allen et al., 2009; Barbosa et al., 2012). This information is processed and integrated by the brain which generates a motor program that selectively activates the expansion/retraction of chromatophores (Figure 1A; Supplementary Figure S1).

A range of environmental stressors might interfere with these neurally controlled mechanisms and result in the alteration of chromatophores’ activity—and, subsequently, of color change. Therefore, the quantitative measurement of physiological color change in cephalopod species could provide new opportunities for toxicological risk assessment. Following a body of research that has evaluated the effect of various environmental stressors on camouflage in juvenile European common cuttlefish, we here discuss the relevance of this species as a toxicological model and address the challenge of color change quantification and standardization through a comparative review of the available measurement techniques.

### 1.1 *Sepia officinalis* as an emerging model in behavioral ecotoxicology

The European common cuttlefish (*Sepia officinalis*) is one of the most studied cephalopod species, especially for the exploration of color change and advanced cognitive functions (Darmaillacq et al., 2014). This necto-benthic species heavily relies on rapid adaptive camouflage to avoid being detected by predators. Cuttlefish are known to exploit a combination of background color matching, disruptive coloration, masquerade and distractive markings, involving more than 30 different chromatic components (Hanlon and Messenger, 1988). Although *S. officinalis* could theoretically produce billions of body patterns, it appears that some combinations of components are preferentially used (Osorio et al., 2022), resulting in the expression of three main camouflage patterns called *uniform*, *mottle* and *disruptive* (Hanlon and Messenger, 2018). Uniform patterns are usually displayed on uniform backgrounds, whereas cuttlefish exhibit more complex patterns such as mottle pattern on sandy bottoms or disruptive pattern on contrasted substrates containing gravel, pebbles or shells. These body patterns are said to be chronic (i.e., maintained over long time periods) as opposed to acute body patterns (i.e., displayed during seconds to minutes for communication, predation or defensive purposes). Their efficiency can be improved by behavioral stillness and sand-digging (Mather, 1986).

The chromatophores of *S. officinalis* are known to be under the control of serotonin, which induces a retraction of the pigment-containing sacculi, and glutamate, which induces their expansion. This antagonistic system would control rapid color changes for the production of acute body patterns, whereas FMRFamide-related peptides and nitric oxide would be involved in the maintenance of chronic patterns by modulating the action of glutamate (Loi and Tublitz, 2000; Mattiello et al., 2010). A proven malfunction of these control systems could therefore reveal a neurotoxic effect or neuroendocrine disruption.

At the time of hatching, the chromatophores are functional and the juveniles are immediately capable of color changes—although their camouflage abilities significantly improve with age (Dickel et al., 2006). This gives the opportunity to implement behavioral tests on the earliest-life stages, with several practical advantages: (1) hatchlings can be available in large numbers from egg clusters collected in the environment, (2) the hatching success is close to 100% and (3) juveniles are relatively easy to maintain in laboratory conditions. Their small size (about 10 mm in dorsal mantle length at hatching against 45 cm for the adults) requires little space for rearing facilities (0.005 m^2^ and 2 L per animal according to Fiorito et al., 2015) and experimental devices. Most importantly, the use of early-life stages is particularly relevant from an ecotoxicological and ecological point of view. Indeed, hatchlings and juveniles are more vulnerable to environmental contaminants than adults, since they are at key stages of their cerebral, cognitive and behavioral development (Dickel et al., 2006). They are also more exposed to contaminants as they spend all embryonic stages and the first post-hatching months in coastal waters subjected to anthropogenic pollutants, before migrating offshore to their wintering grounds (Miramand et al., 2006). In addition, the digestive gland, a key organ for detoxification, has not yet reached its full physiological development in the first month of juvenile life, raising the question of its efficiency to cope with the toxic effects of contaminants (Lacoue-Labarthe et al., 2016).

More generally, the recent interest in using cephalopods in ecotoxicological studies lies in their potential for bioaccumulation (Bustamante et al., 1998b), which should be favored by their short life span and rapid growth (i.e., the accumulation of contaminants followed by reproduction is less energetically expensive than their elimination for survival purpose). In addition, cephalopods are recognized vectors for chemicals transfer along the trophic web as they are predators and prey of numerous marine organisms (Bustamante et al., 1998a). In cuttlefish, bioaccumulation occurs from different uptake pathways, including seawater, sediment (for sand-digging species) and trophic routes (see Bustamante et al., 2002, Bustamante et al., 2004). Consequently, the concentrations of contaminents vary along the environmental conditions affecting bioavailability and physiology (e.g., temperature, *p*CO_2_) and many life traits, such as age, size or diet and trophic levels (for reviews: Lacoue-Labarthe et al., 2016; Penicaud et al., 2017).

To date, neither cuttlefish nor any cephalopod species has been recognized as a model species *per se*. However, *S. officinalis* was acknowledged as an emerging model for research in the fields of cognitive neuro-ethology, sensory ecology and behavioral ecotoxicology (Bassaglia et al., 2013). With an effort toward the harmonization of existing analytical tools and resources, studies devoted to cephalopods could benefit a wider scientific community.

### 1.2 The challenge of color change quantification

The experimental analysis of cuttlefish color change requires (1) properly designing the visual stimuli meant to elicit specific behavioral responses (color change or body pattern) and (2) collecting images of sufficient quality, allowing (3) relevant quantitative image analysis.

To meet the first requirement, fundamental research conducted on cuttlefish camouflage is a valuable resource. While cuttlefish are known to integrate multiple visual features from their surroundings to elaborate a camouflage response, it has been demonstrated that the use of simple artificial substrata can reproducibly elicit typical body patterns. For example, the disruptive body pattern is routinely induced in response to black and white checkerboards displayed on the bottom and walls of the arena. A top requirement is that the checkerboard squares are between 40 and 120% of the size of the animals’ white square component area (see Barbosa et al., 2007). The use of smaller or larger checkerboard squares is likely to induce mottle or uniform body patterns (Chiao and Hanlon, 2001).

The photographs used for color change analyses must also meet certain criteria. The topic was covered by Stevens and collaborators (2007), who emphasize the utmost importance of manually controlling the white balance and light exposure. The lighting should also be tuned so as not to stress tested individuals (<350 lux at water surface according to Fiorito et al., 2015) while being as close as possible to the spectrum of sunlight to offer ecologically relevant testing conditions.

Finally, the camouflage patterns can be characterized using a range of descriptors (see Table 8 three from Josef and Shashar, 2014 for a review). For ecotoxicological purposes, the simple discrimination of uniform, mottle and disruptive patterns can already provide ample information. This can be done manually (Dickel et al., 2006) but also automatically, based on their distinct spatial frequency spectra (Barbosa et al., 2008), or with the help of learning algorithms (Orenstein et al., 2016).

The analysis can be extended to an evaluation of the efficiency of each camouflage. The uniform camouflage pattern can be assessed by a measurement of homochromy, i.e., the matching of the animal mantle compared to the background luminance according to the mean grey value (see Poirier et al., 2005; Di Poï et al., 2014; Supplementary Figure S2). In this case, the standard deviation of the grey values of the animal’s body can also be considered as a descriptor of the heterogeneity of the uniform pattern. Finally, the disruptiveness can be assessed by assigning a score—from 0 (not expressed), 1 (weakly expressed), 2 (moderately expressed) to 3 (strongly expressed)—to each of the eleven chromatic components commonly forming disruptive patterning (Barbosa et al., 2008). Individual cuttlefish can then be assigned a total grade, called disruptive score, ranging from 0, for maximal homochromy, to 33, for highly disruptive patterning (Supplementary Figure S2).

It is also possible to add a temporal dimension to the analyses. It may consist in assessing the latency of a chronic pattern (Court et al., 2022) or its stability over short time periods (Di Poï et al., 2014; Chabenat et al., 2021; Gouveneaux et al., in prep.). Otherwise, the comparison of camouflage quality at different ages during the first months is a simple way to assess whether the typically observed post-embryonic maturation of camouflage abilities is altered (Dickel et al., 2006).

These tests are relatively quick and inexpensive to implement, not to mention that photographic imaging is non-invasive. The reproducibility of image analysis is also facilitated by the fact that cuttlefish usually stay still on the substrate and, therefore, exhibit a portion of its mantle relatively unchanged in size and extension (as opposed to octopodiforms which take on shapes and postures that usually conceal part of their skin). This feature could facilitate the adaptation of more complex quantification methods (e.g., the analysis of grey values profiles along some body axes; Court et al., 2022 adapted from Chiao and Hanion, 2001) to reveal more subtle color change alterations. Indeed, image analysis can be technically challenging and time-consuming, especially if the method is manual and several annotators have to analyze in parallel to avoid bias on the results.

### 1.3 Recent applications in ecotoxicology

A number of environmental stressors have been shown to alter *Sepia officinalis*’ color change abilities. Several studies have focused on the effect of waterborne fluoxetine and venlafaxine, two antidepressants of the class of selective serotonin reuptake inhibitors and serotonin-norepinephrine reuptake inhibitors, respectively (Table 1). Changes were mainly observed in the efficiency of the uniform pattern, which appeared more heterogeneous in animals exposed to one or the other of these compounds (Bidel et al., 2016a, 2016b). However, a mixture of both resulted in a more uniform pattern (i.e., lower disruptive score) when calculated in animals tested on uniform backgrounds (Chabenat et al., 2021). Although venlafaxine did not appear to modulate the serotonin levels in the central nervous system of cuttlefish (Bidel et al., 2016a), it was observed to cause the dose-dependent relaxation of chromatophore-associated muscles in isolated skin patches, as observed in response to serotonin (Gouveneaux et al., in prep).

**TABLE 1:** Summary of uses of body patterns in ecotoxicology (dph: days post-hatching; NE: no effect).

Stressors	Concentrations	Exposure duration	Body patterns	Endpoints	Results	References
Fluoxetine (antidepressant)	1, 10 and 100 ng.L^-1^	15 days pre-hatching to 32 dph	Uniform	*Homochromy percentage Heterogeneity index*	decrease NE	Di Poï et al. (2014); Bidel et al. (2016b)
			Disruptive	*Disruptive score*	NE	
Venlafaxine (antidepressant)	5 and 100 ng.L^-1^	20 dph	Uniform	*Heterogeneity index*	increase	Bidel et al. (2016a)
			Disruptive	*Disruptive score*	NE	
Fluoxetine + venlafaxine	5 ng.L^-1^ fluoxetine 2.5 + 2.5 or 5 + 5 ng.L^-1^ of the mixture	29 dph	Uniform	*Disruptive score*	decrease	Chabenat et al. (2021)
			Disruptive	*Disruptive score*	NE	
Mercury + *p*CO_2_	0,3 and 3 μg.g^-1^dry weight in muscle ∼1,600 µatm	30 dph	Uniform	*Homochromy percentage Heterogeneity index*	NE	Minet (2022)
			Disruptive	*Disruptive score*	light uniform pattern	
Dexmedetomidine (anesthetic, antifouling agent)	10 µg.L^-1^	3 dph	Uniform	*Homochromy percentage Heterogeneity index*	dark uniform pattern	Gouveneaux et al., in prep
			Disruptive	*Disruptive score*	dark uniform pattern	

More recently, these tests have been adapted to monitor the effects associated with trophic mercury, a potent neurotoxin under its methylated form (MeHg) (Minet, 2022; Figure 1B), as well as the effects of suspected neuroendocrine disruptors (i.e., dexmedetomidine) (Gouveneaux et al., in prep). In contrast to previously cited studies, juvenile cuttlefish were shown to struggle mostly with the disruptive pattern: the chronic exposure to MeHg resulted in the display of a light uniform pattern on black and white checkerboards. In contrast, acute exposure to the antifouling agent dexmedetomidine resulted in a dark uniform pattern regardless of the type of background presented (light or dark grey uniform backgrounds, black and white checkerboards; Figure 1C). Finally, seawater carbon dioxide (*p*CO_2_) is of growing interest due to its effects on nervous system and behaviors in cephalopods (e.g., Spady et al., 2018).

It must be noted that, while one-month-old juveniles exposed to elevated *p*CO_2_ (∼1,600 µatm) failed to produce a disruptive pattern on the black and white checkerboards (Minet, 2022), exposure of hatchlings to ∼1,000 µatm did not affect the camouflage display as assessed with a different quantification method regardless of the substrate nature (Court et al., 2022). This raises the question of how the animals’ response depends on its age, the contaminants’ pressure levels and the descriptors used for color patterns. In sum, this emphasizes the need to harmonize the camouflage assays.

## 2 Discussion

Cephalopods combine the advantages of being keystone species in marine food webs, important fishing and aquaculture resources, and valuable experimental animals. As such, they stand at the crossroads of several of the environmental problems of the Anthropocene. As a matter of fact, human activities have substantially changed the world’s marine environments in recent decades. While some populations and species are paying a high price for these changes, up to the point of collapse or extinction. Cephalopods, however, seem to take advantage of these major changes (Doubleday et al., 2016). This success is partly attributed to the “live fast, die young” life history strategies of cephalopods, which confer them great plasticity and adaptability to environmental changes (O’Brien et al., 2018). However, these overall trends of populations can mask a continuous decrease in the European cuttlefish stocks in the last decade (FAO, 2019), suggesting that this species may be vulnerable to multiple, co-acting stressors (Clarke, 1996; Bustamante et al., 1998b). Therefore, their study might shed light on our understanding and assessment of the risks associated with pollutants and related stresses in marine environments.

In this perspective, the quantification of physiological color change in cuttlefish is emerging as an easily accessible, non-invasive, sensitive and holistic ecotoxicological endpoint. Indeed, color change determines camouflage, whose efficiency depends on (1) visual abilities and acuity (from eye functioning to pigments structure), (2) information processing (nervous system) and (3) control of chromatophore dynamics (nervous and hormonal control pathways) (see Reiter and Laurent, 2020) with which many contaminants can interfere. Previous studies, notably on fish, crustaceans and cephalopods (e.g., Lennquist et al., 2010; Ford and Feuerhelm, 2020; Chabenat et al., 2021), have highlighted various effects of contaminants on color change, ranging from impaired acclimatization abilities to changing visual environments up to persistent darkness or paleness. The underlying causes of these alterations remain poorly understood, although in most cases they are likely explained by the disruption of color change control mechanisms. It appears so for venlafaxine, whose topical effects on *S. officinalis* (i.e., the concentration of pigments and skin paling), are consistent with its mode of action, namely, an increase in serotonin levels (Gouveneaux et al., in prep).

Yet, unusual mantle color changes or discoloration are commonly used in cephalopods—although quantitative assessment methods are rarely implemented—as indicators of anesthesia or general ill-being (Polese et al., 2014; Fiorito et al., 2015). As it generally goes with behavior, this emphasizes that color change is the expression of an integrated physiological state and carries the potential to reveal a wide spectrum of disruptions beyond those affecting the chromatophore control mechanisms themselves. This points to the possibility of confounding factors, which exist for many toxicological endpoints. Thus, the assessment of color change must be completed by sensory tests (such as visual acuity assessment; see Cartron et al., 2013) but also neurotransmitters quantification or genomic approaches, to highlight neurotoxic effects.

The use of *S. officinalis* as a model proved to be convenient, with fundamental studies about its color change offering a solid knowledge base and a wide range of analytical tools. However, scoring methods, such as the disruptive score, are still time-consuming and experimenter-dependent with a qualitative interpretation of the expression of chromatic components. Thus, one of the main challenges in the development of cuttlefish color change as a proxy for ecotoxicology will be the development of automatized and, above all, standardized methods to ensure the reproducibility and comparability of tests. This applies to the methods of image acquisition and analysis, but also to the conditions of exposure to contaminants, as well as the age of the animals tested. Finally, it is essential to develop positive controls, i.e., to use chemical compounds with known modes of action, such as neuromodulators, to characterize and mark out the nature and magnitude of effects that can be expected on cuttlefish color changes.

Most studies conducted so far have focused on the description of chronic body patterns and their components. However, a variety of subtle color changes and body patterns, including the repertoire of acute body patterns displayed by *S. officinalis*, remain to be assessed under stressful conditions for possible derivation as toxicological endpoints. Besides, finer scale studies could lead to a better understanding of the hierarchical organization of chromatophores and thus of the impact of contaminants on neuronal activity. This could rely on existing methods for automatic analysis of chromatophores activity, which have significantly improved (Goodwin and Tublitz, 2013; Hadjisolomou and El-Haddad, 2017). In fact, it is currently possible, using deep learning algorithms, to perform automatic delineation and tracking of individual chromatophores, to classify them as light or dark and to discriminate overlapping structures (https://git.unicaen.fr/nicolas.elie/redpol-open). Such a tool was recently developed from video recordings of *S. officinalis’* skin explants, following the bioassay protocol implemented by Loi and collaborators (1996) for highlighting the pharmacological basis of chromatophore control. It will likely be able to process high-resolution images acquired from motile cuttlefish, as recent works have shown (Reiter et al., 2018; Hadjisolomou et al., 2021). Yet, the number of animals needed for *ex vivo/in vitro* tests is reduced by the potential for high-throughput using of several skin patches from one animal only.

In conclusion, color change appears to be an integrated endpoint of the subtle effects of contaminants that can interact at different processing levels (visual perception, neuro-hormonal control, motor dynamic of chromatophores, or even decision-making) and thus provide early warning information of the cuttlefish vulnerability to environmental stressors. In turn, improving the knowledge on the toxicodynamics of contaminants targeting specific pathways would help understand the complex biological processes governing camouflage. Finally, the ecological consequences of affected camouflage abilities on cuttlefish fitness remain to be explored.

## Data Availability

The raw data supporting the conclusion of this article will be made available by the authors, without undue reservation.
