# Supplementary material for: Cuttlefish color change as an emerging proxy for ecotoxicology
Source: Front Physiol. 2023 Mar 8;14:1162709. doi: 10.3389/fphys.2023.1162709 (PMC10030679; doi:10.3389/fphys.2023.1162709)
Supplement: Supplementary file 1 [file DataSheet1.docx]

# Supplementary Material


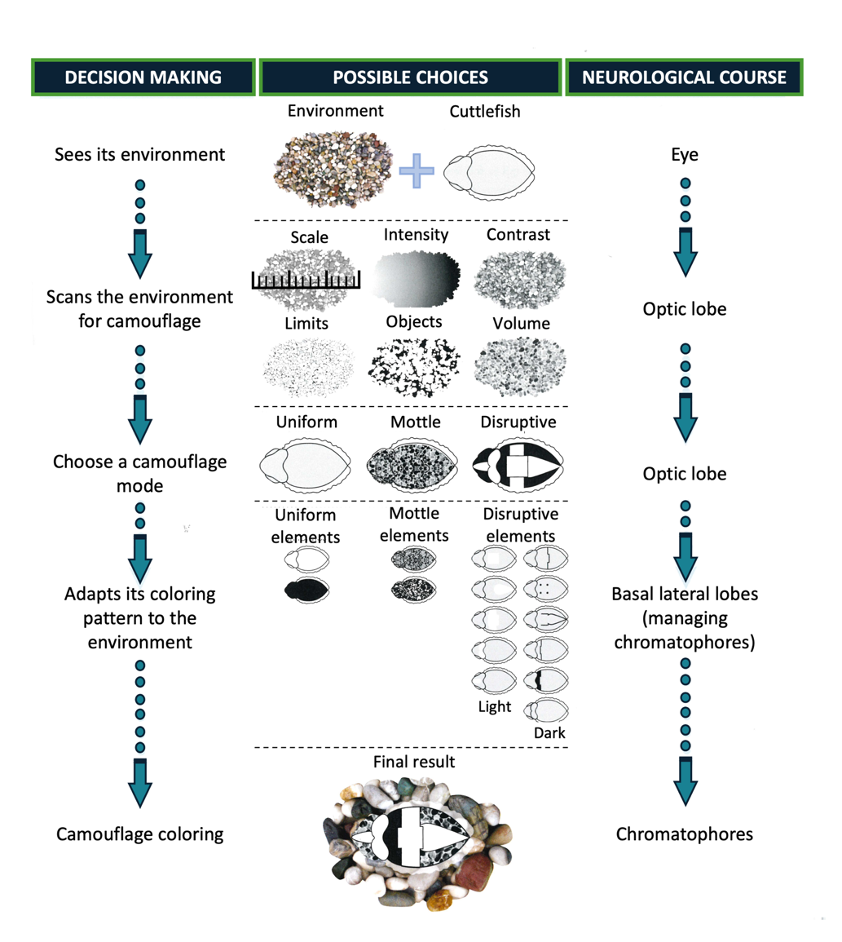


**Figure S1.** Cuttlefish camouflage decision processing with decision making, possible choices and neurological course (adapted from Hanlon et al., 2019).


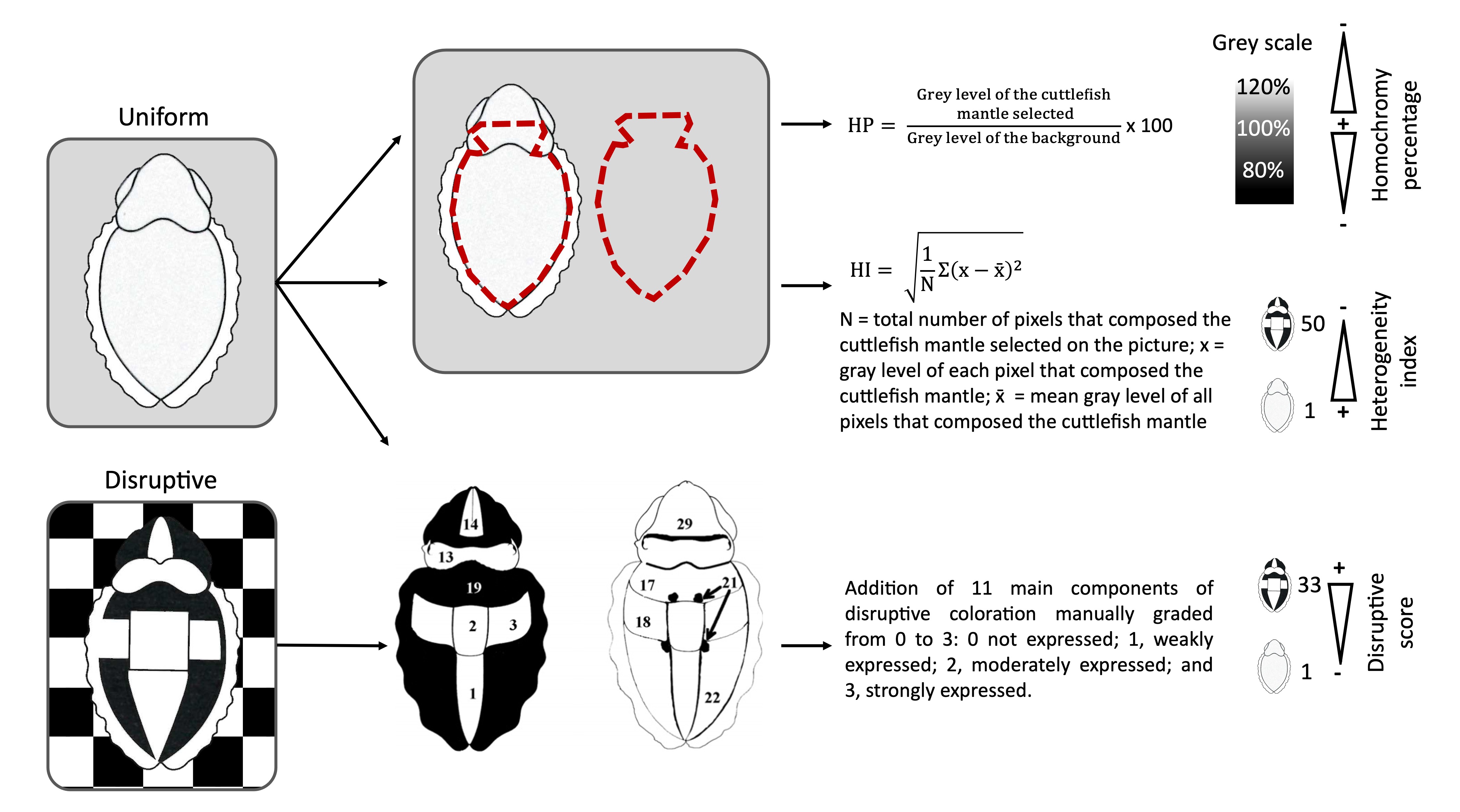


**Figure S2.** Summary of the most used scoring methods to evaluate cuttlefish uniform and disruptive body patterns: Homochromy percentage (HP), heterogeneity index (HI) and disruptive score (DS)..
